# Comparative Effects of Dietary Supplementations with Microencapsulated Sodium Butyrate, Glycerol Monolaurate and Tributyrin on Growth, Immunity, and Gut Health in Black Sea Bream

**DOI:** 10.3390/ani15060810

**Published:** 2025-03-12

**Authors:** Sami Ullah, Fengqin Feng, Minjie Zhao, Jinzhi Zhang, Qingjun Shao

**Affiliations:** 1 Zhejiang University Zhongyuan Institute, Zhengzhou 450001, China; samiwazir04@yahoo.com (S.U.); fengfq@zju.edu.cn (F.F.); 2 College of Biosystems Engineering and Food Science, Zhejiang University, Hangzhou 310058, China; minjiezhao@zju.edu.cn; 3 College of Animal Sciences, Zhejiang University, Hangzhou 310058, China

**Keywords:** *Acanthopagrus schlegelii*, glycerol monolaurate, microencapsulated sodium butyrate, tributyrin, growth performance, gene expression, microflora

## Abstract

This study investigates the effects of three dietary additives—microencapsulated sodium butyrate (MSB), glycerol monolaurate (GML), and tributyrin (TB)—on the growth performance, various physiological parameters, gene expression, intestinal morphology, and microflora in *Acanthopagrus schlegelii* (black sea bream). The experiment utilized a 43.5% soybean meal (SBM) inclusion diet with four isonitrogenous and isoenergetic formulations: a control diet, and diets supplemented with MSB (0.24%), GML (0.04%), or TB (0.22%). The growth trial spanned eight weeks, and triplicate tanks were randomly assigned to each diet, with each tank containing 30 fish, each having an initial weight of 1.55 ± 0.01 g. Key outcomes included measurements of weight gain, specific growth rate, digestive enzyme activity, serum immune markers, antioxidant status, and intestinal morphology and, gut microbiota. Additionally, gene expression and microbiota analysis were conducted on intestinal tissues to assess the impact of these additives on gut health and immune response. The findings revealed that all three additives enhanced growth performance and improved intestinal health and gut microbiota but GML exhibited the most pronounced effects on intestinal barrier function and immune modulation, gene expression, and microflora, followed by MSB and TB. This study provides a comprehensive comparison of MSB, GML, and TB as feed additives for black sea bream, offering insights into their potential for improving fish health and optimizing aquaculture feed formulations.

## Simple Summary

This study investigated the comparative effects of microencapsulated sodium butyrate, glycerol monolaurate and tributyrin on the growth, digestive enzyme activity, immune function, blood antioxidant levels, and intestinal structure of black sea bream. Additionally, incorporating MSB, GML, and TB into the diet resulted in significant improvements in villus height, crypt depth, the villus-height-to-crypt-depth ratio, and the number of goblet cells per unit of villus height in the anterior section of the intestine. However, in this study, we also checked gene expression and microflora. The present results indicate that GML is highly beneficial, with a 0.04% dosage being the most effective as compared to MSB (0.24%), and TB (0.22%) in our study. The results could contribute to the development of healthier aquatic species, potentially lowering both the costs and environmental impact of aquaculture. These findings indicate that microencapsulated sodium butyrate, glycerol monolaurate, and tributyrin may be ideal dietary supplements for fish.

## 1. Introduction

The aquaculture industry has exploded in recent decades, owing in part to the rising demand for human food [1]. The aquaculture sector is one of the emerging sectors with an 8% average yearly growth rate. Fisheries and aquaculture provide almost 20% of protein worldwide and it is estimated that worldwide it provides 3.2 billion fish [1]. According to the 2024 edition of *The State of World Fisheries and Aquaculture* (SOFIA) [2], global fisheries and aquaculture production reached 223.2 million tonnes in 2022, representing a 4.4% increase compared to 2020. This total comprised 185.4 million tonnes of aquatic animals and 37.8 million tonnes of algae. This growth can be attributed to advancements in various areas, including production techniques, nutritional approaches, disease management, and genetic improvements. A critical area of focus in fish nutrition and pathology is intestinal health, which includes the microbiome composition, structural integrity, and immune function. Asia is recognized as a leading global contributor to aquaculture production. The aquaculture industry mainly runs on fish feed. Humans can obtain much of their necessary minerals, proteins and vitamins from fish [3]. It offers over 20% of the world’s seafood protein to approximately 3.2 billion people [1]. Fish require good quality food to enhance their growth and maintain good health. So a certain level of protein and essential additives in the feed of fish are regularly added [4]. By improving disease resistance, growth, survival rates and the quality of fish feed, medicine and production expenses can be reduced [5]. Previously, much research has been carried out on the formulas, ingredients and methodologies of feed [6]. For instance, fatty acids have recently been identified as a source of energy and performing a number of functions, including metabolic regulation, anti-inflammatory effects, and antimicrobial activity [7]. The three types of fatty acids mainly include short-chain fatty acids (SCFAs), medium-chain fatty acids (MCFAs), as well as long-chain fatty acids (LCFAs). Short-chain fatty acids and LCFAs are recognized as playing a key role in enterocyte energy metabolism. Short-chain fatty acids are a class of fatty acids characterized by aliphatic tails containing one to six carbon atoms, the most prevalent of which are acetic (C2), propionic (C3), and butyric (C4) acids, whereas MCFAs are fatty acids with seven to twelve carbon atoms. Long chain fatty acids are derived from number twelve. In feed, SCFAs are being studied as an alternative to antibiotics [8]. In mammals, SCFAs are produced by anaerobic bacterial fermentation of non-starch polysaccharide and unabsorbed starch [9].

SCFAs mainly include butyric, propionic, as well as acetic acids which have been shown to have a prominent role in improving the metabolic rate in fish species [10]. For instance, butyric acid (butanoic acid), one of the SCFAs, has a number of important roles in the digestive system. The enteric cells immediately absorb and utilize the butyric anion that works as a main source of fish energy. It also stimulates intestine paracellular permeability reduction, mucosal cell proliferation, pathogen inhibition, intestinal immune response regulation, intestinal cell development, and intestinal barrier function improvement [11]. Because naturally produced butyrate levels in the colon and caecum are low [12], sodium butyrate is utilized as a good substitute instead of butyric acid in feed to produce butyrate which is less odorous, and has solid and stable qualities [13]. In swine mucosa cells, sodium butyrate boosted antioxidant indices while decreasing malondialdehyde (MDA) levels [14]. Carp [15] and tilapia [15] grew faster with sodium butyrate in their meals [16]. Sodium butyrate, a fat-soluble molecule, was shown to be readily absorbed and used as a source of energy by enteric epithelial cells [15]. Butyrate’s pharmacological properties, including its short half-life and hepatic clearance in the first pass, reduce its ability to circulate in the system [17]. Moreover, to achieve the needed concentrations, high dosages of butyrate are frequently required [18]. The effects of non-coated butyrate have only been studied in the upper gastrointestinal system [19]. To solve the disadvantages of non-coated sodium butyrate (SB), a microencapsulated sodium butyrate (MSB) needed to be developed. MSB is a better option than ordinary SB in this circumstance for achieving long-term SB release into the intestine. Butyrate is released slowly into the gastrointestinal tract by microencapsulated sodium butyrate, allowing it to enter the large intestine [15]. Song et al. [20] investigated the impact of food supplementation with MSB in broiler chickens and found evidence of improvement in intestinal barrier issues and growth performance that had previously been afflicted with necrotic enteritis. According to Liu et al. [15], the immunological response and intestinal condition of common carp are improved by oral supplementation with dietary MSB.

Medium-chain fatty acids have been developed as promising alternatives due to their unique physiological properties and role as energy sources. These fatty acids demonstrate potential as effective feed additives, presenting a viable substitute for antibiotics [21]. Their antimicrobial properties have attracted significant interest, largely attributed to their natural presence in foods such as coconut oil and distinctive metabolic pathways [22]. Consequently, ongoing research is investigating the application of MCFAs as a potential lipid replacement in animal feed [23]. Medium-chain fatty acids are efficiently absorbed from the gastrointestinal tract and transported directly to the liver via the portal vein. Conversely, LCFAs are mainly absorbed via the lymphatic system before reaching the systemic circulation [24]. In contrast to LCFAs, MCFAs are absorbed by cells without the need for membrane transporters and are directly delivered to the mitochondrial intermembrane space, eliminating the requirement for the carnitine shuttle. Studies have shown that monoglycerides of MCFAs, especially glycerol monolaurate (GML), exhibit antipathogenic activity. As a representative medium-chain monoglyceride, GML is readily digestible, effectively absorbed, and demonstrates potent antioxidant properties [21].

Glycerol monolaurate (GML), is natural lauric acid glycerol monoester obtained from coconut oil. GML is a natural food emulsifier according to the US Food and Drug Administration. It has no maximum dosage in food products, with amounts ranging from 10 mg kg^−1^ to 2000 mg kg^−1^ in foodstuffs and health care products (21 CFR GRAS 182.4505). GML inhibits the growth and pathogenicity of a number of fungi, bacteria, and enveloped viruses in vitro, in addition to emulsification [25]. GMLs are increasingly being employed in ordinary foods such as cereals, meats, and soft drinks accordingly, because of these factors [26]. As a result, GML is a superb antimicrobial emulsifier that is widely used in commercial applications and consumed by the general public on a regular basis. Glycerol monolaurate is a surfactant found naturally in oil of coconut that is extensively used as a food emulsifier and antibacterial agent [27]. Immunomodulatory effects of GML have recently been found [26]. Glycerol monolaurate is an antibacterial and naturally occurring fatty acid molecule [28]. Numerous Gram-negative and Gram-positive bacteria, viruses, and enveloped fungi are inhibited in their growth and pathogenicity by glycerol monolaurate [25]. Glycerol monolaurate has a lengthy residence period in the gastrointestinal system and so comes into direct contact with the gut microbiota, which has major effects on physiology and host health, particularly in metabolism and immunological development [29]. It is now widely acknowledged that interactions between gene products and microbial cell components such as peptidoglycan, lipopolysaccharides (LPS), and flagellin have a long-term effect on the host’s metabolism and immune system [30].

Among the SCFAs, tributyrin (TB) (C_15_H_26_O_6_), produced from butyrate, has recently attracted a lot of attention in nutrition research for its role in helping animals use the nutrients in their food more efficiently. Glycerin is esterified with an excess of butyric acid to produce tributyrin [31]. This feed additive was discovered to be a good source of butyrate, which is required for improved growth performance as well as the maintenance and development of the gastrointestinal system [32]. So far, the results of utilizing TB in fish have been inconclusive. According to prior research, 0.24% microencapsulated sodium butyrate with a 36% SBM diet [33], glycerol monolaurate 0.04% with a 43.50% SBM diet [34], and tributyrin 0.22% with a 45.00% SBM diet [35] were appropriate doses for optimum growth in black seam bream.

*A. schlegelii* (black sea bream) is a warm water fish found along China’s south-east coast and in other Asian countries [36]. It is currently cultured in Korea, China, Japan and Southeast Asian regions [36]. Furthermore, black sea bream is a valued commercial marine fish in China, as well as Japan, Taiwan, Korea, and Southeast Asia, due to its mild, white meat, which is considered among the best of any white-meat fish. The goal of this study was to compare MSB, GML, and TB to explore the dietary effects on different aspects of growth performance, proximate composition, digestive enzymes, serum immunity, antioxidant parameters, gene expression, intestinal development, and microflora in black sea bream. Our study will offer a theoretical basis for the future use of MSB, GML, and TB in further improving black sea bream growth performance in the future for industry purposes.

## 2. Materials and Methods

### 2.1. Formulation and Composition of Diets

The microencapsulated sodium butyrate (MSB) was provided by Hangzhou King Technica, glycerol monolaurate (GML), and tributyrin (TB) materials were obtained from the South China University of Technology. As the control (0.00), an isonitrogenous (41.5%) and isoenergetic (19.0 kJ/g) diet was developed based on the nutritional requirements of black sea bream. The remaining three dietary treatments were supplemented with either 0.20% microencapsulated sodium butyrate (MSB), 0.04% glycerol monolaurate (GML), or 0.20% tributyrin (TB). The primary protein sources consisted of fishmeal, soybean meal, and soy protein concentrate while the main lipid sources included fish oil, corn oil, and soybean lecithin (Table 1). The ingredients were ground and mixed thoroughly before adding oil and water to form a homogeneous dough. The dough was then extruded to produce pellets, which were air-dried and stored at −20 °C until use.

### 2.2. Animal Management, Experimental Location and Parameters

Juvenile black seabream was sourced from the Marine Fisheries Research Institute in Zhejiang, China. The experimental trials were conducted at the Joint Laboratory of Nutrition and Feed for Marine Fish, part of the Zhejiang Marine Fisheries Research Institute, located on Xixuan Island, Zhoushan. Prior to the growth trial, the fish were temporarily housed in an indoor concrete tank (10 m × 4 m × 2 m) and acclimatized for two weeks, during which they were fed a commercial diet daily (41.5% crude protein level). Following this acclimation period, 360 fingerlings with an initial body weight (IBW) of 1.55 ± 0.01 g and age of 42 days were randomly distributed into eighteen cylindrical fiberglass containers (350 L capacity), with 30 fish per container. Three replicate tanks were assigned to each dietary treatment at random. The aquarium was haphazardly given to the treatment groups in triplicates and for eight weeks nourishment was provided 3 times daily (8 am, 12 pm and 16 pm) to superficial satiety. The seawater supply was processed by pumping it from the ocean and allowing it to settle in a sedimentation pool for 48 h before use. Afterward, the seawater was filtered through a sand filter before being supplied to each tank. The seawater temperature was naturally relatively stable at 27 ± 1 °C and the flow rate was about 2 L min^−1^. Tanks were aerated continuously with the help of air stones and the dissolved oxygen was ≥5.0 mg L^−1^. The multiparameter was used to check the following parameters. The salinity was sustained at 28 ± 2 g L^−1^, the pH ranged within 8.1–8.3. Light/dark was kept to twelve hours. Tanks were cleaned one hour after the final feeding, based on the breeding environment. Fecal collection commenced in the sixth week of the growth trial. Fecal samples were routinely collected at 6:00 am. according to the method described by Bureau et al. [37] and were stored at −20 °C for preservation.

### 2.3. Sample Collection

After 8 weeks, the fish were subjected to a 24 h feed deprivation period, followed by anesthesia using an MS-222 solution at a concentration of 60 mg L⁻^1^. Subsequently, specific growth rate (SGR), weight gain (WG), and survival rate were recorded. Stocking density was also monitored to ensure appropriate feed management.

For proximate analysis of the fish samples, 3 fish were randomly selected per aquarium, preserved at −20 °C. Blood, required for serum, was taken with 1 mL gauge syringe from the dorsal side of the body, followed by 15 min centrifugation (equipped with thermostat at 4 °C) at 4500 rpm for serum extraction, and preserved then at −80 °C. While the other dissected parts were removed carefully to obtain the hepatosomatic, viscera somatic index, and intra-peritoneal fat ratio, and were weighed.

The intestine was the primary target organ in this study, and tissue samples were collected as follows:

Three whole, emptied intestines were collected from each tank and stored at −80 °C for subsequent gene expression analysis. Each intestinal organ from the aquarium was segmented into two distinct sections: anterior and posterior. Approximately 3 cm of each intestinal segment was excised, rinsed, and cleaned with chilled phosphate-buffered saline (PBS, pH 7.0) for subsequent morphological analysis, followed by fixation in 10% formalin. Furthermore, 3 cm segments of each intestine were preserved in PBS containing 2.5% glutaraldehyde and stored at −4 °C for subsequent scanning electron microscopy (SEM) analysis.

For enzymatic activity analysis, the remaining intestines were stored till analysis, at −20 °C.

### 2.4. Sample Analytical Methods

The fish samples were subjected to proximate analysis using the methods outlined by the Association of Official Analytical Chemists (AOAC) [38].

Determination of moisture content: keeping the samples at 105 °C (time:1 day)

Content of ash determination: burning of samples for one day (approx. 550 °C).

Determination of nitrogen content: (N × 6.25): Kjeldahl method was used to analyze crude protein using Kjeldahl system (Model; semiauto; KDN-20C; Zhejiang TOP Instrument Co., Ltd., Hangzhou, China)

Determination of crude lipid using Soxhlet for 6 h.

### 2.5. Digestive Enzyme Activity

Gut samples were separated and mixed with ice-cold physiological saline (weight by volume 0.85%) followed by thermostat-centrifugation at 6000× *g* (20 min). Supernatants obtained were examined for TP, protease, amylase and lipase, using Nanjing Jincheng Bioengineering Institute (Nanjing, China) diagnostic reagent kits.

### 2.6. Serum Parameters

Using assay kit obtained from Nanjing Jincheng Bioengineering Institute (Nanjing, Jiangsu Province, China), the total concentrations of protein (TP), malondialdehyde (MDA), antioxidant capacity (T-AOC), superoxide dismutase (SOD), albumin (ALB), catalase (CAT), glutathione peroxidase (GSH-Px), aspartate aminotransferase (AST), alanine transaminase (ALT), lysozyme (LZM), immunoglobulin M (IgM), complement protein C3, cholesterol (T-CHO), and C4 were measured in the serum. Following are the manipulation procedures, performed as per manufacturer’s instructions.

### 2.7. Intestinal Histology

#### 2.7.1. H&E Staining Sample

The removal of water from the preserved samples was conducted in graded sequences of ethanol and cleared with the help of xylene. After that, tissues were fixed in paraffin wax, cut into 5 μm pieces and then hematoxylin/eosin (HE) was used for staining the tissues. Using OLYMPUS (CX21) equipped with Motic Digi Lab II software, photomicrographs of the sections were observed. The following variables were measured: villi height, VH; crypt depth; CD, and amount of GC, goblet cells with software, Image Pro-Plus (6.0) (Media-Cybernetics, Inc., Tokyo, Japan)).

#### 2.7.2. Scanning Electron Microscopy (SEM) Examination

The gut samples which were preserved previously underwent rinsing in a mixture of glutaraldehyde and 0.1 M PBS (pH 7.0), followed by post-fixation with 1% osmium tetroxide (OsO4) for a duration of 1–2 h. The samples were then subjected to a graded ethanol dehydration series: 30%, 50%, 70%, 80%, 90%, 95%, and finally 100% pure ethanol. Following dehydration, the samples were placed in a 1:1 mixture of isoamyl acetate and alcohol for 30 min, and subsequently stored overnight in pure isoamyl acetate. Before observation, dehydration was performed in a critical point dryer (Hitachi Model HCP-2) utilizing liquid CO_2_ for 4–5 min. The samples were then coated with a gold-palladium layer using a Hitachi Model E-1010 ion sputter, Tokyo, Japan. Observations were conducted utilizing a Hitachi Model TM-1000 scanning electron microscope (SEM).

Microvilli density was measured using SEM photomicrographs captured at 35,000× magnification, with three images analyzed per dietary treatment. Microvilli were identified based on their characteristic round shape in the images. The number of microvilli was counted within a 1 μm^2^ area, with three areas chosen per image. Image-Pro Plus 6.0 (Media Cybernetics, Inc., Tokyo, Japan) software was used for the analysis.

### 2.8. Analysis of Gene Expression

#### RNA Extraction and Quantitative Real-Time PCR Analysis (qRT-PCR)

Total RNA was extracted using Trizol reagent (Vazyme Biotech Co., Ltd., Nanjing, China). The RNA concentration and quality were assessed using a NanoDrop 2000 spectrophotometer (Thermo Fisher Scientific, Wilmington, DE, USA) and 1% agarose gel electrophoresis. RNA samples with an OD260/280 ratio between 1.8 and 2.0 and an OD260/230 ratio ≥ 2.0 were selected for cDNA synthesis. Reverse transcription was performed using the PrimeScript™ RT Reagent Kit with gDNA Eraser (TaKaRa Company of Biotechnology, Dalian, China). Specific primers for qRT-PCR were designed using Primer Premier 5.0 software, with nucleotide sequences obtained from the NCBI database (https://www.ncbi.nlm.nih.gov/) and previous studies. Primer stability was evaluated via 1% agarose gel electrophoresis, and their specificity was validated following the protocols described by Chang, et al. [39]. The synthesized cDNA was diluted (1:5) in DEPC-treated water and combined with primers and TB Green^®^ Premix Ex Taq™ (Takara, China) for qRT-PCR, which was performed using a LightCycler 480 system (Roche, Basel, Switzerland). The qRT-PCR thermal cycling conditions included an initial denaturation at 95 °C for 1 min, followed by 45 cycles of denaturation (5 s), annealing at 60 °C (15 s), and extension at 72 °C (20 s), with a final melting curve analysis and cooling to 37 °C.

All RT-qPCR reactions were conducted in triplicate, The expression levels of insulin-like growth factor I (IGF-I), growth hormone (GH), cholecystokinin (CCK), interleukin-1 (IL-1), transforming growth factor (TGF), claudin-1 (CLDN-1), caspase-3 (CAS-3), and G protein-coupled receptor 41 (GPR41) were quantified utilizing the 2^−ΔΔCT^ method [40] and normalized against β-actin. Each sample was analyzed in triplicate by RT-qPCR. The genes IGF-I, GH, CCK, IL-1, TGF, CCK, CLDN-1, CAS-3, and GPR41 were obtained from the NCBI website (http://www.ncbi.nlm.nih.gov), and primers were designed using Primer (5.0) software, as detailed in Table 2.

### 2.9. Microbiota Analysis

The procedures for DNA extraction, PCR amplification, and Illumina MiSeq sequencing were conducted as outlined below. Genomic DNA was extracted from fecal samples (n = 4) utilizing the E.Z.N.A.^®^ Stool DNA Kit (D4015, Omega Bio-tek, Wilmington, DE USA). PCR amplification of the V4–V5 regions of the bacterial 16S rRNA gene was conducted utilizing universal primers 338F (5′ ACTCCTACGGGAGGCAGCAG 3′) and 806R (5′ GGACTACHVGGGTWTCTAAT 3′). The PCR reaction mixture comprised 12.5 µL of Phusion Hot Start Flex 2X Master Mix, 2.5 µL of each primer, and 50 ng of template DNA. The PCR amplification products were resolved through electrophoresis on a 2% agarose gel, followed by the extraction of target fragments utilizing the AxyPrep PCR Cleanup Kit. The pooled library was subsequently loaded onto the Illumina MiSeq platform for paired-end sequencing (2 × 300 bp) utilizing the MiSeq Reagent Kit V3 (600 cycles).

### 2.10. Statistical Analysis and Calculation

The equations outlined below were employed to assess growth performance and feed utilization.

Initial average body weight (IBW, g)

Final average body weight (FBW, g).

Weight gain rate (WGR, %) = 100 × (final body weight − initial bodyweight)/initial body weight

Specific growth rate (SGR, %/day) = 100 × (ln final body weight − ln initial body weight)/days.

Mean feed intake (MFI, g fish^−1^ d^−1^) = air dry fed in g/(fish in g × day)

Feed conversion ratio (FCR) = dry feed weight (g)/wet weight gain (g)

Protein efficiency ratio (PER) = wet weight gain (g)/total protein intake (g).

Condition factor (CF, g cm^−3^) = 100 × [(final body weight in g)/(final body length in cm)^3^]

Hepatosomatic index (HSI, %) = 100 × (liver weight in g/body weight in g).

Intraperitoneal fat ratio (IPR %) = 100 × (intraperitoneal fat weight in g/body weight in g).

Viscerosomatic index (VSI, %) = 100 × (viscera weight/body weight).

Protein productive value (PPV, %) = 100 × protein gain (g)/total protein intake (g).

Survival rate (SR, %) = 100 × (final fish number/initial fish number)

Data were analyzed using a one-way analysis of variance (ANOVA), followed by Tukey’s post hoc test for pairwise comparisons. The results are presented as means ± standard error (SE), with statistical significance determined at *p* < 0.05. Statistical analyses were conducted using SPSS software (Version 22.0) and the graph was created using OriginPro 24 software.

## 3. Results

### 3.1. Growth Performance and Feed Utilization

The results of growth performance and feed utilization are summarized in Table 3. Across all dietary treatments, the survival rate (SR) exceeded 99.3%, indicating no significant negative impact on survival from the supplemented diets. The final body weight (FBW), weight gain (WG), and specific growth rate (SGR) were significantly higher (*p* < 0.05) in fish fed the MSB, GML, and TB diets compared to the control group. No significant differences were observed in the mean feed intake (MFI), feed conversion ratio (FCR), condition factor (CF), hepatosomatic index (HSI), intraperitoneal fat ratio (IPF), and visceral somatic index (VSI) across the treatments (*p* > 0.05). The protein efficiency ratio (PER) and protein productive value (PPV) were notably improved in the supplemented groups compared to the control diet (*p* < 0.05). These results suggest that dietary supplementation with MSB, GML, and TB enhances growth performance without affecting the morphometric parameters or feed efficiency (*p >* 0.05).

### 3.2. Composition of Whole Body and Dorsal Muscle

Table 4 summarizes the proximate composition of the whole body and dorsal muscle after the feeding trial. There were no significant differences in moisture, lipid, ash, or protein content between the control and experimental groups for both the dorsal muscle and whole body (*p* > 0.05). However, the protein content in the whole body in the treated groups and the ash content in the dorsal muscle was significantly higher in the control group compared to the supplemented groups (*p* < 0.05). This lack of significant changes in nutrient composition aligns with previous studies on other species, suggesting that the dietary inclusion of MSB, GML, and TB does not drastically alter body composition.

### 3.3. Digestive Enzyme Activity

The digestive enzyme activity results are shown in Table 5. No significant differences were observed in the activities of total protease (TP), trypsin, lipase, or amylase between the experimental groups (*p* > 0.05). This suggests that the supplemented diets had no adverse effect on the digestive capacity of the fish, consistent with findings in other species where enzyme activity remained unaffected despite dietary modifications.

### 3.4. Serum Biochemical, Immune and Antioxidant Parameters

The immune and antioxidant parameters of serum are presented in Table 6. There was no effect on the antioxidant and immune parameters in the serum of TP, ALB, SOD, MDA, T-AOC, LZM, ALT, AST, T-CHO, IgM, C3 and C4 (*p* > 0.05) in any of the treatment groups in the serum. However, GSH-Px, and CAT levels were significantly higher in the GML group, as compared to the other groups (*p <* 0.05).

### 3.5. Biochemical, Immune, and Antioxidant Parameters in the Hindgut and Midgut

The immunological and antioxidant parameters of fish hindgut and midgut are shown in Table 7. The TP, LZM, ALB, IgM in hindgut, and TP, ALB, T-AOC, and IgM in midgut were not affected (*p >* 0.05) in any of the groups. However, T-AOC in control and TB groups were significantly lower, compared to GML, and MSB in hindgut, and the LZM level in midgut was significantly lower in the control, MSB, and TB as compared to GML (*p <* 0.05).

### 3.6. Biochemical, Immune, and Antioxidant Parameters in the Liver

The antioxidant and immune parameters of the liver are shown in Table 8. The TP, SOD, GSH-Px, CAT, T-AOC, and AST of the liver showed no changes with treatments (*p >* 0.05). ALT was significantly lower, compared with the GML group. However, the MDA contents showed a descending trend.

### 3.7. Liver Gene Expression

**Figure 1:**
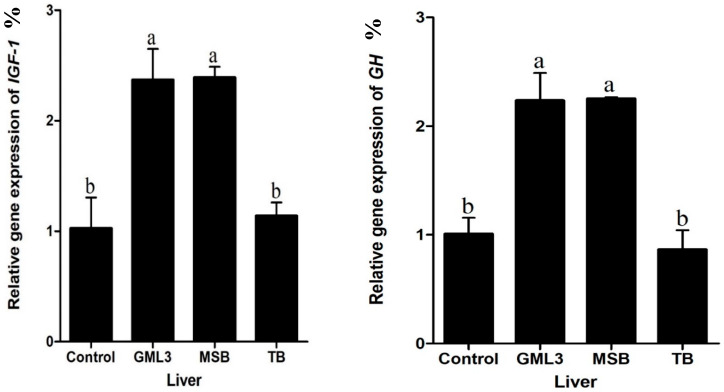
Relative expression levels of hepatic insulin-like growth factor-1 (IGF-1) and growth hormone (GH) genes in fish fed control, GML, MSB, and TB diets. Different letters on bars show significant difference (*p* < 0.05). a, b letters indicate statistical significance between the diet groups. a vs. b show a significant difference.

IGF-1 and GH gene expression are shown in Figure 1. Both IGF-1 and GH were significantly lower than control, and TB, compared to the GML and MSB groups (*p* < 0.05).

### 3.8. Intestinal Gene Expression

**Figure 2:**
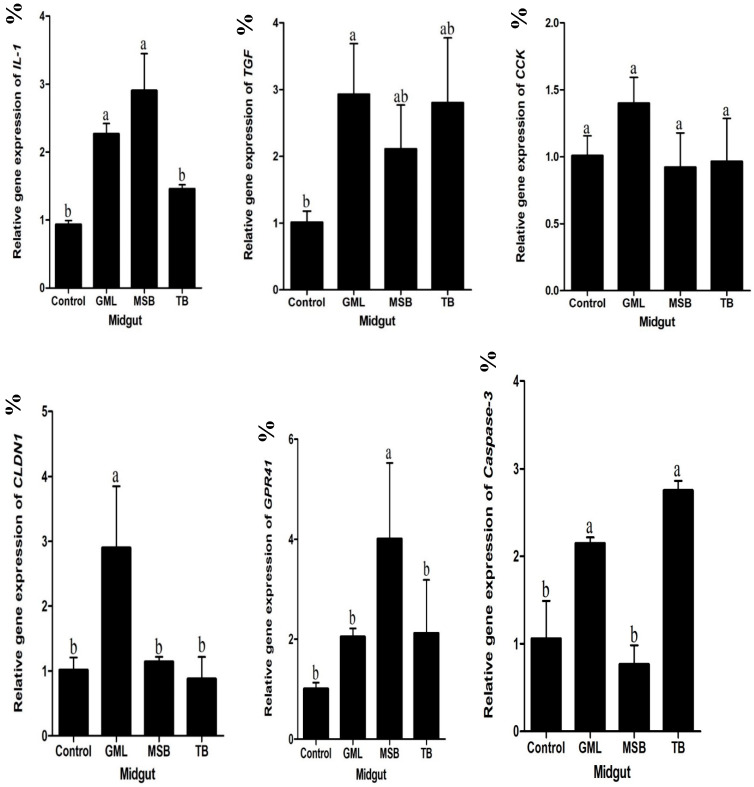
Relative expression levels of interleukin-1 (IL-1), transforming growth factor (TGF), cholecystokinin (CCK), claudin-1 (CLDN1), caspase-3 (CAS-3), and G protein-coupled receptor 41 (GPR41) genes in the midgut of fish fed control, GML, MSB, and TB diets. Different letters on bars show significant difference (*p* < 0.05). a, ab, b letters indicate statistical significance between the diet groups. a and ab are not significantly different, while a vs. b show a significant difference.

Figure 2 shows the expression of intestinal immune and growth-related genes. The pro-inflammatory cytokine interleukin-1 (IL-1) was significantly lower in the control group and TB (*p* < 0.05) as compared to the MSB and GML groups, while transforming growth factor (TGF) expression was significantly lower in the control group compared to the GML group (*p* < 0.05), suggesting a role in maintaining gut integrity and immune balance. Cholecystokinin (CCK) gene expression was non significantly high in all groups but enhanced in the GML group. Claudin-1 (CLDN1), a tight junction protein, was also significantly upregulated in the GML group, supporting improved gut barrier function. Caspase-3 (CAS-3), a marker of apoptosis, was significantly higher in the GML and TB groups compared to the MSB and control groups (*p* < 0.05), indicating potential regulatory effects on intestinal cell turnover. Additionally, the short-chain fatty acid receptor GPR41 was highly expressed in the MSB group, indicating potential interactions between the diet and gut microbial activity.

### 3.9. Intestinal Histology Structure and Morphometric Parameters

**Figure 3:**
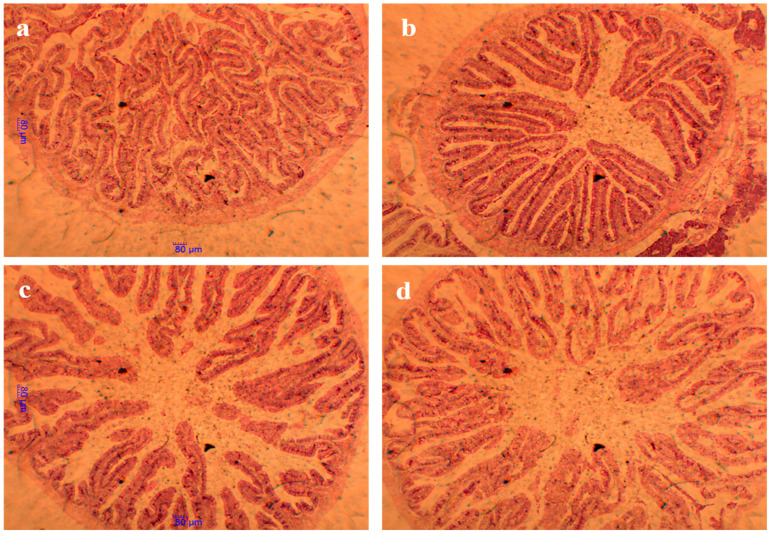
Histology (H&E) of foregut villus structure of *A. schlegelii* fed the experimental diets (×40). Note: (**a**) fish fed control diet; (**b**) fish fed MSB diet; (**c**) fish fed GML diet; (**d**) fish fed TB diet. Photo (**a**) indicated condensed villus height (VH), fewer goblet cells (GC), and smaller villus surface area (VSA); photos (**b**–**d**) had longer villi and more goblet cells.

**Figure 4:**
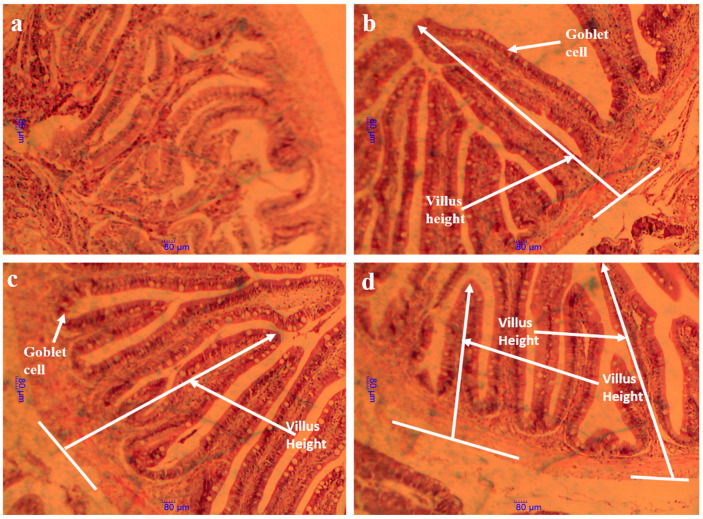
Histology (H&E) of foregut villus structure of *A. schlegelii* fed the experimental diets (×100). Notes: (**a**) fish fed control diet; (**b**) fish fed MSB diet; (**c**) fish fed GML diet and (**d**) fish fed TB diet. Photo (**a**) indicated condensed villus height (VH), fewer goblet cells (GC), and smaller villus surface area (VSA); photos (**b**,**c**,**d**)had longer villi and more goblet cells.

**Figure 5:**
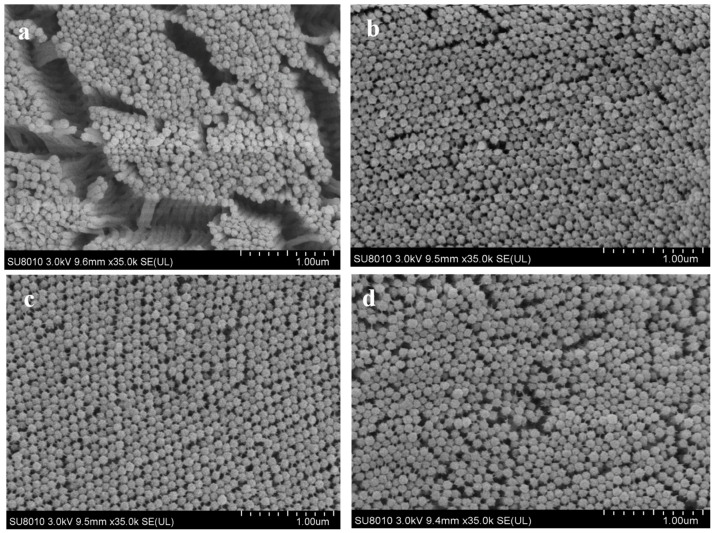
Effect of dietary MSB, GML and TB on foregut microstructure of juvenile fish (×5000). Notes: (**a**) control group; (**b**) MSB group; (**c**) GML group; (**d**) TB group.

The results of the intestinal morphometric parameters are presented in Table 9. The light microscope photomicrographs of the foregut section of the fish are shown in Figure 3 (×40) and Figure 4 (×100), while SEM photomicrographs of the foregut section are shown in Figure 5. Multiple signs of damage were found in the guts of fish fed a high soybean-based control diet, including a significant reduction in villus height (VH), crypt depth (CD), villus height/crypt depth ratio (VH/CD) ratio, and finally a reduction in the number of goblet cells/villus height (GC/VH). MSB, GML, and TB had significantly greater VH, CD, VH/CD, and GC/VH ratios than controls (*p >* 0.05). Fish given MSB, GML, or TB diets had normal morphological structure, including intact villus folds (Figure 3a–d and Figure 4a–d) and a broad absorptive surface brimming with microvillus (Figure 3a–d and Figure 4a–d).

### 3.10. Intestine Histological Structure

**Figure 6:**
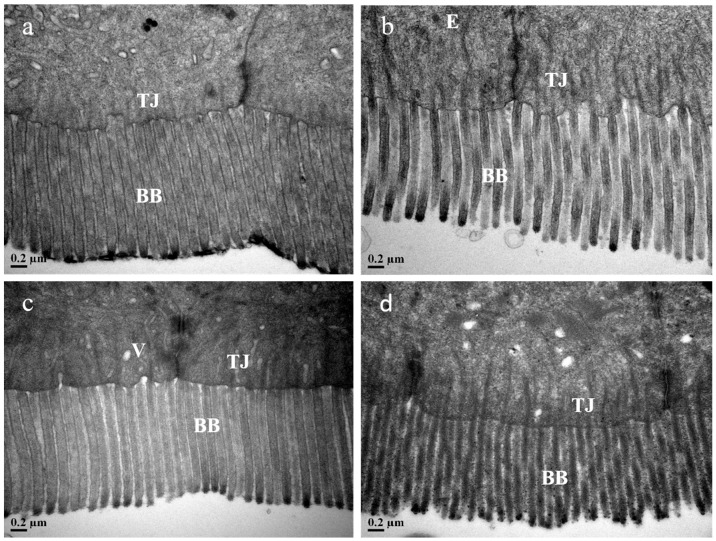
TEM photomicrographs of the foregut of fish fed with experimental diets (0.2 μm). Notes: (**a**) control; (**b**) MSB; (**c**) GML; (**d**) TB. Transmission electron microscopy, scale bar, 0.2 μm; BB, brush border; TJ, tight junction; E, enterocyte; V, vacuole.

**Figure 7:**
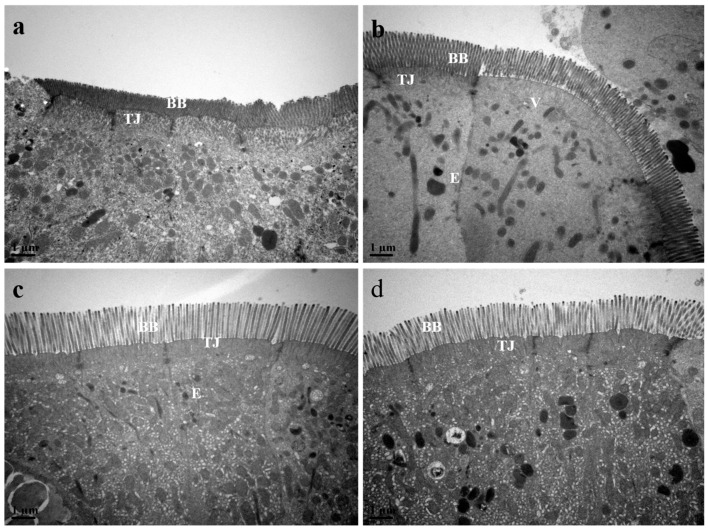
TEM photomicrographs of the foregut of fish fed with experimental diets (1.0 μm). Notes: (**a**) control; (**b**) MSB; (**c**) GML; (**d**) TB. Transmission electron microscopy, scale bar, 1.0 μm; BB, brush border; TJ, tight junction; E, enterocyte; V, vacuole.

The TEM photomicrographs of the fish foregut are presented in Figure 6 (0.2 μm) and Figure 7 (1.0 μm). All fish from the various dietary groups exhibited a foregut characterized by a columnar epithelium composed of tall cells, featuring well-defined and organized brush borders. The MSB, GML, and TB groups exhibited longer brush borders in comparison to the control group. The cells appeared to be connected by a complete junctional complex, with no observable intercellular spaces.

### 3.11. Analysis of the Composition and Relative Abundance of the Microbial Community

#### 3.11.1. Overview of Sequencing Data for Intestinal Microbiota

The intestinal microbiota sequencing data are presented in Table 10. A total of 1,299,371 raw tags were obtained, averaging 82,465 tags per sample, with individual sample counts ranging from 80,000 to 86,000. After quality filtering, the dataset was refined to 1,167,396 clean tags, with an average of 74,913.25 tags per sample and a range spanning from 69,974 to 80,000. Taxonomic units representing a variety of bacterial species were identified from the pool of clean tags, with all samples exhibiting Good’s coverage values surpassing 0.999.

#### 3.11.2. Alpha Diversity and Coverage Analysis of the Intestinal Microbiota

Dietary interventions had a significant impact on richness estimates (Chao1) and diversity measures, such as the Simpson and Shannon indices. A total of 223 operational taxonomic units (OTUs) were identified within the intestinal microbiota across all four groups analyzed. The GML fish group showed the highest abundance of unique OTUs, whereas the control group had the lowest, as presented in Table 11.

#### 3.11.3. Phylum Level

**Figure 8:**
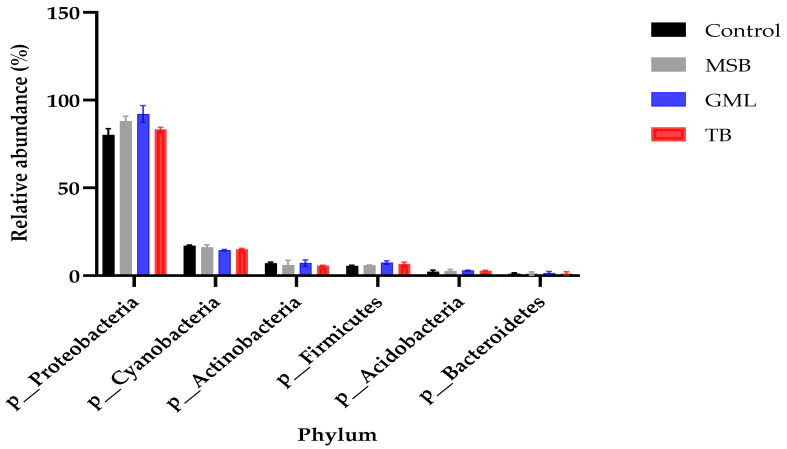
The relative abundance of gut microbiota at the phylum level in black sea bream (n = 4 per group).

The intestinal microbiota composition of black sea bream was analyzed at the phylum level. Six distinct phyla were identified across the fish exposed to different dietary treatments. The Proteobacteria was the most abundant phylum, followed by Cyanobacteria, Actinobacteria, Firmicutes, Acidobacteria, and Bacteroidetes in descending order. The abundance of Actinobacteria, Acidobacteria, and Bacteroidetes did not show significant changes (*p* > 0.05) in response to the dietary treatments. The treated groups exhibited a significant increase in the abundance of Proteobacteria, Cyanobacteria, and Firmicutes when compared to the control group (*p* < 0.05) (Table 12) and (Figure 8).

#### 3.11.4. Class Level

**Figure 9:**
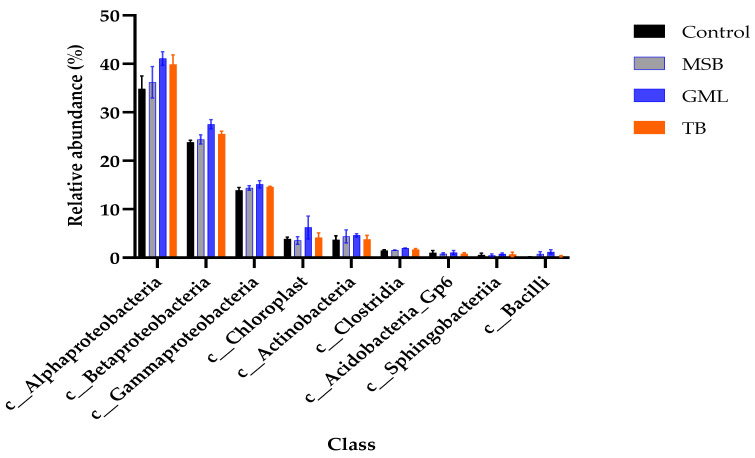
The relative abundance of gut microbiota at the class level in black sea bream (n = 4 per group).

The microbiota composition was analyzed at the class level across different dietary groups. Nine unique classes were detected in the intestines of fish subjected to the dietary treatments. Alphaproteobacteria was the highly abundant class, followed by Betaproteobacteria, Gammaproteobacteria, Chloroplast, Actinobacteria, Clostridia, Acidobacteria_Gp6, Sphingobacteriia, and Bacilli in descending order. The abundance of Chloroplast, Actinobacteria, Acidobacteria_Gp6, and Sphingobacteriia was not significantly affected (*p* > 0.05) by the dietary treatments. Overall, the abundance of Alphaproteobacteria, Betaproteobacteria, Gammaproteobacteria, Clostridia, and Bacilli increased in the supplemented groups compared to the control group (*p* < 0.05) (Table 13) (Figure 9).

#### 3.11.5. Family Level

**Figure 10:**
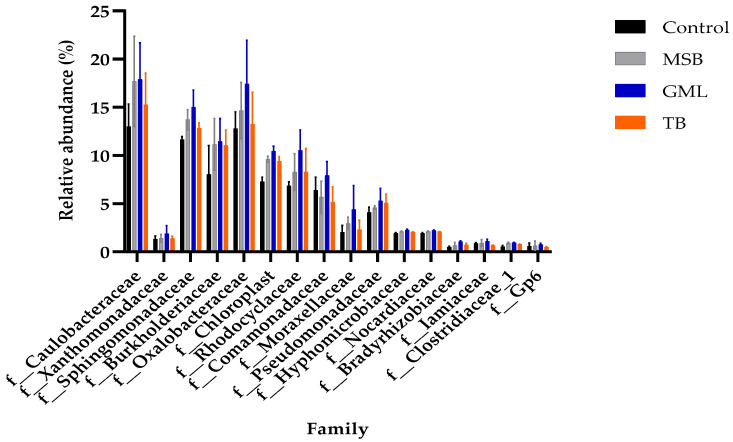
The relative abundance of gut microbiota at the family level in black sea bream (n = 4 per group).

The composition of the microbiota at the family level is presented in Table 14 and illustrated in Figure 10. A total of 16 families were identified in the intestines of fish that underwent dietary treatments. The abundance of families such as Caulobacteraceae, Xanthomonadaceae, Burkholderiaceae, Oxalobacteraceae, Rhodocyclaceae, Comamonadaceae, Moraxellaceae, Pseudomonadaceae, and Gp6 was not significantly altered (*p* > 0.05) across the dietary groups. However, the abundance of Sphingomonadaceae, Chloroplast, Hyphomicrobiaceae, Nocardiaceae, Bradyrhizobiaceae, Iamiaceae, and Clostridiaceae_1 significantly increased with dietary supplementation (*p* < 0.05).

## 4. Discussion

Diets enriched with fatty acids (FAs) offer advantages over other feeds due to their rapid digestibility and ability to be passively absorbed by animals [41]. Short-chain fatty acids, particularly butyric acid (butanoic acid), are believed to perform numerous beneficial functions in the gastrointestinal tract of animals. These include promoting the development of intestinal cells, inhibiting pathogens, stimulating mucosal cell proliferation, supporting growth, modulating intestinal immunity, and enhancing intestinal barrier function [11,42]. Medium-chain fatty acids, particularly glycerol monolaurate (GML) is well-known for its diverse properties, functioning as an antifungal, antiviral, anti-inflammatory, and antibacterial agent [43]. The inclusion of antimicrobial drugs in chicken feed has historically resulted in various negative outcomes, including changes in intestinal microbiota, the presence of drug residues in meat and eggs, environmental contamination, and the development of antibiotic-resistant microorganisms [44]. Given the rising public concern over the health risks linked with excessive antibiotic use in animal feed, there is an increasing demand for exploring natural alternatives. Similarly, the inclusion of organic acids in the diet has been shown to promote fish growth. This effect is directly influenced by factors such as the type of organic acid used, the composition of the diet, the fish species, as well as the age and farming conditions of the fish [45].

In the present study, MSB, GML and TB supplements were compared with the control group as sources of fatty acids to improve the growth performance of fish in a high SBM level diet. Meanwhile, MSB, GML and TB treatments were numerically higher than the control treatment. Our study revealed that incorporating fatty acids into feed enhanced weight gain, improved overall growth performance, and boosted the specific growth rate in juvenile black sea bream. Comparable results were reported by Hong et al. [46], where dietary supplementation with caprylic and capric acids improved growth performance in pigs. These findings suggest that both free medium-chain fatty acids (MCFAs) and MCFAs bound to triglycerides in piglet diets significantly improved body weight gain and feed efficiency compared to a control group fed soybean oil [46]. Furthermore, research involving pigs demonstrated that GML holds substantial potential as a growth promoter and as an alternative to antibiotics in animal nutrition [47], consistent with the results of our study. Our study demonstrated that the inclusion of glycerol monolaurate (GML) in the diet positively influenced the growth of black sea bream. This aligns with previous research indicating that medium-chain fatty acids can promote growth in juvenile common sea bream [15], tilapia [16], and crucian carp [48]. In this study, feed intake was not affected, which is consistent with our findings, as reported in tilapia [49]. This suggests that the inhibitory effect on feed intake may vary based on the fish species and the source of dietary fatty acids (FAs). The protein efficiency ratio (PER) results indicate that incorporating glycerol monolaurate (GML) into the diet significantly improves protein utilization in black sea bream. These findings are consistent with previous studies reported by [50]. Additionally, research has identified the liver as the primary site for the metabolism of fatty acids following their absorption and digestion [51]. Furthermore, no significant differences in the intraperitoneal fat ratio (IPR) were observed between the groups with a supplemented diet and the control group, aligning with earlier findings in grass carp [52]. MSB, GML, and TB were compared to the control group as sources of fatty acids in order to improve fish development performance in a high SBM diet in the present study. According to [53], adding MCT to a sow’s food improved suckling piglets’ jejunum villus height and daily body weight gain. Lactating sows’ feed intake was not improved by fatty acids [54]. These findings are also related to our study.

The proximate compositions of the fishes’ whole-body and dorsal muscle showed no adequate modification after dietary treatments in the current study, and so our findings are consistent with observations on *Arctic charr* and grass carp [52], as well as in the non-significance observed in the GML, MSB, and TB treatments in fish [33,34,55].

Digestive enzymes are critical for the breakdown and absorption of nutrients, thereby enhancing weight gain and overall health in fish. Evaluating the activity of these enzymes provides insights into the nutrient assimilation efficiency of specific diets [56]. In our study, dietary interventions involving TP, trypsin, lipase, and amylase demonstrated no significant variations in activity, aligning with earlier findings. According to [34], they also obtained the same results. Furthermore, supplementation with fatty acids and *Cuphea* seed components was associated with increased villus height in piglets [29].

An animal’s primary defence mechanisms against oxidative stress are xenobiotic-produced antioxidant enzymes [57]. Antioxidant enzymes such as SOD, GSH-Px, and CAT protect animals from injury. Superoxide radicals (HO_2_/O_2_) are dismutated to the less dangerous H_2_O_2_, and H_2_O_2_ is detoxified to H_2_O and O_2_ by GSH-Px and CAT [58]. The antioxidant effects of feed additives have been found to improve overall gut function [59]. The most significant content by which to measure the health of living beings is serum biochemical properties, as previously stated [60]. Changes in blood parameters are caused by unbalanced diets and nutritional deficits [61]. Previous MSB, GML, and TB research has primarily focused on the microbiota of animals, with limited information on the effects on immunological and hematological markers. Dietary addition of protexin and SB was shown to impact growth performance immunological response and the blood parameters of *Oreochromis niloticus* [16]. The growth performance, immunological response, and blood parameters were all significantly improved in this study. In this study, we analyzed total protein (TP), albumin (ALB)), glutathione peroxidase (GSH-Px), malondialdehyde (MDA) catalase (CAT), total antioxidant capacity (T-AOC), superoxide dismutase (SOD), complement component 3 (C3), lysozyme (LZM), alanine transaminase (ALT), immunoglobulin M (IgM), total cholesterol (T-CHO), complement component 4 (C4), and aspartate aminotransferase (AST). All serum immune and antioxidant markers showed positive improvements. These findings are consistent with the results of a previous study by Ullah et al. [33]. The elevated TP levels observed in this study indicate enhanced protein metabolism [62]. Moreover, a reduction in liver MDA levels was noted, which aligns with the findings of [63], where the antioxidant effects of citric and malic acids were reported in a poultry study.

Plasmas globulin, released by B cells after they have been transformed into the plasma cells, increases when the level of antibodies rises. The lower ratio of globulin/globulin in blood, that measures the immune state, suggests that the synthesis of globulin has increased and the immunity has been enhanced. And the presence of a high level of total blood proteins indicates that the intestines and liver were involved in making sufficient protein [64]. The ALT level in the liver, GHS-Px and CAT were found to be significantly higher in this investigation. In vitro, butyrate has been demonstrated to boost the activity of antioxidant enzymes. According to Jahns et al. [65], butyrate exposure dramatically boosts the antioxidant enzymes in the normal colon cells. After four days of butyrate therapy, the SOD enzymatic activity is enhancing in Caco-2 colon cancer cells [66]. Fatty acid has been demonstrated to have a positive effect on SOD and GSH-Px in swine mucosa cells, which is surprising [14]. It was also reported that grass carp liver showed an increase in SOD and GSH-Px, but no change in MDA activity following fatty acid administration [7]. Anti-inflammatory and antibacterial properties, as well as the capacity to improve immunity and prevent swine reproductive abnormalities, are all features of fatty acids [67]. Studies have also indicated that fatty acid dietary supplementation also increases the IgM levels. According to Fang et al. [68], who added SB to weaned swine diets, an increase in the plasma IgM content was achieved by adding MCFA and SCFA to diets [69].

In this study, we investigated the effects of MSB, GML, and TB supplementation on the health and nutritional intake of black sea bream fingerlings. Various biochemical and antioxidant parameters of the liver were evaluated. The findings showed that supplementation with different fatty acids did not significantly affect the physiological and immune parameters of the treated fish groups. These results are consistent with those reported in a previous study [70]. Similar results were observed in previous studies that involved lauric acid supplementation [55].

Various genes play pivotal roles in fish growth, with particular emphasis on growth hormone receptor and as well as growth factor genes, such as insulin-like growth factor-1 [71]. Growth factors are generated through endocrine stimulation by growth hormone (GH) or through paracrine/autocrine signaling mechanisms in target tissues. The GH-IGF axis is the main pathway that regulates growth and tissue proliferation in most vertebrates. In the present study, the expression of growth hormone receptor genes, including GH, and growth factor genes, specifically IGF-1, was significantly upregulated in response to MSB and GML supplementation. These findings are consistent with previous research [72]. The liver is identified as the principal site for IGF synthesis in fish, with paracrine secretion occurring in muscle tissues.

In Gram-positive bacteria, the lipoteichoic acid (LTA), a component of the cell wall, acts as a ligand for Toll-like receptor-2 (TLR-2), triggering a cascade of events that include the release of cytokines such as interleukin-1 (IL-1) [73]. In black sea bream, supplementation with GML has been shown to upregulate the expression of IL-1, and transforming growth factor (TGF). The same results were also aligned with [70]. Existing literature highlights that the expression of proinflammatory cytokines, such as interleukin-1 (IL-1), and transforming growth factor (TGF), can be effectively influenced by dietary interventions in various fish species [74]. Previous studies have reported an increase in the expression of proinflammatory cytokine genes, including IL-1, and TGF, following probiotic supplementation. In our study, treated groups resulted in a significant upregulation of these anti-inflammatory cytokines, specifically IL-1, and TGF. Previous studies have documented a comparatively higher expression of pro-inflammatory cytokine genes in tilapia following feeding [75]. Supporting this, tilapia showed upregulated expression of interleukin-1 (IL-1) genes, which correlated with enhanced disease resistance [76]. Similarly, the upregulation of IL-1 gene expression observed in our study indicates the activation of effective anti-inflammatory signaling pathways. This conclusion is reinforced by a study that reported an increased expression of interleukin-1 (IL-1) in the intestines of *O. niloticus* after feeding [77]. Consistent with this, our findings show significant elevations in serum lipopolysaccharide (LPS) levels, as well as pro-inflammatory cytokines such as IL-1 and TGF in the treated group (Figure 2), indicating that different fatty acids induce a pronounced state of systemic low-grade inflammation. Cholecystokinin (CCK) is crucial for regulating digestive functions and plays a key role in appetite regulation, particularly in signaling meal termination in vertebrates. In the current study, a notable increase in CCK levels was observed, aligning with prior research conducted in the intestine of ballan wrasse [78]. Claudins are integral membrane proteins found in the tight junctions of epithelial and endothelial tissues, where they are critical for maintaining barrier integrity and regulating inflammation. In our study, a significant upregulation of CLDN-1 was observed, consistent with findings reported in a North American cohort [79]. While caspase-3 genes have been extensively studied in vertebrates, their functions in aquatic animals remain less well understood, highlighting the need for further research in this area [80]. The caspase 3 family is considered a vital immune gene in both vertebrates and invertebrates [81]. The complexities of fish tissue pose challenges in fully understanding how factors that influence apoptotic stimuli contribute to the activation of apoptosis and impact mitochondrial mechanisms during postmortem storage of fish. While extensive research has focused on the structure, properties, and functions of caspase-3 across different species, its role in oysters has also been investigated [81]. This study demonstrated an increase in caspase-3 levels following a GML-supplemented diet, consistent with similar findings reported in *Eriocheir sinensis* [82]. GPR41, a G protein-coupled receptor (GPCR) present in mammals, is expressed in a variety of human tissues, including peripheral blood mononuclear cells, adipocytes, and colon epithelial cells. The increased expression of GPR41 noted in the MSB-supplemented groups suggests its potential importance in the intestine. Furthermore, GPR41 has been detected in adipose tissue in mice, where it has demonstrated the ability to stimulate leptin secretion [83].

In animals, intestinal villi play a vital role in the digestion and absorption of nutrients while simultaneously supporting immune function by serving as a habitat for microbiota [84]. The intestinal villi are composed of epithelial cells that facilitate digestion and nutrient absorption, while goblet cells produce mucus to protect the underlying layers and ensure lubrication for the movement of food materials. Dietary supplements, such as soybean meal, have been shown to enhance intestinal integrity [85]. Furthermore, research suggests that fatty acids (MCFAs), including lauric acid, act as direct energy sources for enterocytes [55]. Aligned with previous findings, the present study demonstrates that GML positively affects the structural properties of the intestinal mucosa in juvenile black sea bream. Previous studies demonstrated that the diets of fish enhance intestinal function in juvenile common carp (*Cyprinus carpio*) [15], catfish (*Clarias gariepinus*) [86], tilapia (*Oreochromis niloticus*) [16], and goldfish (*Carassius auratus*) [87]. Diets supplemented with MSB, GML, and TB were found to be more effective than control diets in controlling the immunological function of black sea bream. The ultrastructure micrograph of the black sea bream gut showed a similar brush border structure in all dietary groups compared to the control group. Decreased crypt depth and increased intestinal villus height are both advantageous to nutrient absorption in general [88]. Generally, the presence of vacuoles in the enterocytes is a normal processing mechanism for good digestion, absorption and transport of nutrients; however, the over-accumulation of these vacuoles may affect the functional alterations of the enterocytes [89]. Moreover, the large intercellular space between enterocytes was reported to be associated with a certain pathological condition in mammals [90]. Fatty acids can assist and enhance the function and morphological structure of the intestines to minimize intestinal mucosal cell damage caused by pathogenic intestinal bacteria through sterilization or bacteriostatic agents [7]. Our results showed the alteration associated with the presence of cell vacuolization in the intestinal enterocytes in the control group. However, this effect was not significant because the microvillus cell showed a long brush border with a tight junction which helped with the transfer of nutrients in the cell. This finding indicated that the actions of fatty acid supplementation were probably more efficient in terms of intestinal cell integrity compared to control supplementation in a gut ultrastructure examination. More investigation is suggested to confirm this finding.

In animals, the gut microbiota is essential for sustaining overall health and physiological balance. The diversity and composition of intestinal microbiota in fish are determined by microbial competition within the intestinal ecosystem for available nutrients and space. Maintaining a balanced intestinal microecology is crucial for promoting optimal fish growth, which relies on a diverse population of beneficial intestinal bacteria. This diversity is influenced by factors such as life stage, diet, and both abiotic and biotic conditions in the culture environment [91]. Fatty acids exhibit notable antibacterial properties, contributing to the stabilization of the intestinal microbiota balance in animals [92]. In this study, the alpha diversity indices of the intestinal microbiota in black sea bream (Simpson, Shannon, Chao1, and observed species) were significantly impacted, suggesting that the inclusion of fatty acids affects the overall microbial diversity and richness. These findings are consistent with the results reported by Ullah et al. [70]. Contrary to our findings, dietary supplementation with berberine at suboptimal carbohydrate levels was reported to enhance the intestinal microbiota diversity in blunt snout bream [93].

In our study, the GML-treated groups exhibited a marked increase in the relative abundance of Firmicutes, Cyanobacteria, and Proteobacteria at the phylum level, emphasizing GML’s capacity to change the microbiota composition at the taxonomic tier. At phylum level, the dominant intestinal microbiota in *Trachinotus ovatus* primarily comprised Proteobacteria, consistent with findings from previous studies [94]. Essential beneficial microflora, such as Proteobacteria, Firmicutes, and Bacteroidetes, are known to produce extracellular digestive enzymes. These enzymes facilitate the breakdown of food into minor molecules, enhancing utilization, and nutrient absorption within the fish gut [95]. As noted by Ghanbari et al. [96], Proteobacteria, Cyanobacteria, and Firmicutes collectively account for up to 90% of the intestinal microbiota phyla in various fish species. The phyla Cyanobacteria, Acidobacteria, Firmicutes, Proteobacteria, Actinobacteria, and Bacteroidetes were identified in the dietary groups. However, the supplementation of fatty acids resulted in elevated concentrations of Proteobacteria, Cyanobacteria, and Firmicutes. Our results are consistent with previous studies in fish species, which reported that Proteobacteria, Cyanobacteria, and Firmicutes are the most dominant microbial phyla in fish gut. The abundance of these phyla was unaffected by diet [97]. The elevated abundance of Cyanobacteria in the GML groups may reflect conditions that promote their proliferation, potentially supporting a balanced microbial diversity within the microbiota. In this study, the treated groups demonstrated a higher abundance of Proteobacteria, Cyanobacteria, and Firmicutes compared to the control group. Our results are consistent with previous studies on fish species, which identified Proteobacteria and Firmicutes as the predominant microbial phyla in the fish gut, regardless of diet [97]. Notably, Firmicutes, accounting for approximately 70% of the microbial population, represent a transient community in the distal intestinal segment [97]. These bacteria are widely acknowledged for their beneficial roles and are frequently utilized as probiotics in vertebrates, particularly in fish. Our findings reveal that dietary supplementation with fatty acids significantly enhanced gut microbiota abundance compared to a control diet, consistent with similar observations reported in previous studies [55].

In the current study, Alphaproteobacteria, Betaproteobacteria, Gammaproteobacteria, Clostridia, and Bacilli demonstrated significantly higher abundance compared to the control group, suggesting that fatty acid supplementation has the potential to modulate the microbiota composition at the class level. Our results are consistent with the findings of [55]. The increased abundance of Gammaproteobacteria indicates their potential role in cellulose degradation and their adaptation to plant-based diets. A similar pattern was observed in previous studies on fish following dietary supplementation with SILOhealth 108Z [98]. Notably, Kollanoor et al. [99] reported that caprylic acid and its monoglyceride component, present in both short- and medium-chain 1-monoglycerides of the SILOhealth 108Z mixture, exhibited in vitro antibacterial activity against *Edwardsiella* species within the Gammaproteobacteria class. Our findings align with the effects of GML treatment on gut microbiota, highlighting its potential influence on host physiology, and health, particularly in relation to metabolism and immune system development [29].

In the current study, Caulobacteraceae, Xanthomonadaceae, Burkholderiaceae, Oxalobacteraceae, Rhodocyclaceae, Comamonadaceae, Moraxellaceae, Pseudomonadaceae, and Gp6 were not affected in any groups, This result is consistent with the findings of Ullah et al. [55]. Sphingomonadaceae, Chloroplast, Hyphomicrobiaceae, Nocardiaceae, Bradyrhizobiaceae, Iamiaceae, and Clostridiaceae_1 demonstrated significantly higher abundance compared to the control group, suggesting that GML supplementation has the potential to modulate the microbiota composition at the class level. Our findings are consistent with prior studies on the effects of lauric acid treatment on gut microflora [55]. Treatment with lauric acid, coupled with modifications in gut microbiota, exerts a considerable impact on host health and physiological functions, especially in aspects like metabolic processes and immune system development [100].

## 5. Conclusions

In summary, the current study suggests that an appropriate level of dietary MSB (0.24%), GML (0.04%), or TB (0.22%) in a high SBM-based diet may improve growth performance, immune response, and intestinal mucosal structure. But the most significant is GML when compared to MSB, and TB. The present results indicate that GML is highly beneficial, with a 0.04% dosage being the most effective as compared to MSB (0.24%), and TB (0.22%) in our study. Additionally, the inclusion of GML in the diet resulted in significant enhancements in villus height, crypt depth, villus-height-to-crypt-depth ratio, and the number of goblet cells per unit of villus height in the anterior intestinal region. However, GML also affected the microflora as compared to MSB, and TB. They offer good results for aquaculture, such as improved diet formulations leading to healthier fish and reduced disease risk, reducing antibiotic dependence, promoting more sustainable fish farming. To better understand the molecular mechanisms underlying these effects, further molecular-based investigations are recommended.

## Figures and Tables

**Table 1:** Ingredients and nutrient composition of experimental diets (%).

Ingredient	Diets			
	Control	MSB	GML	TB
FM	19.9	19.9	19.9	19.9
SBM	43.5	36.0	43.5	45.0
Soy protein concentrate	4.0	4.0	4.0	4.0
Soy lecithin	2.0	2.0	2.0	2.0
Corn oil	6.4	6.4	6.4	6.4
Fish oil	3.0	3.0	3.0	3.0
α-starch	7.0	7.0	7.0	7.0
Squid liver meal	3.0	3.0	3.0	3.0
MSB	0.0	0.24	0.0	0.0
GML	0.0	0.0	0.04	0.0
TB	0.0	0.0	0.0	0.22
Ca(H_2_PO_4_)_2_·H_2_O	2.5	2.5	2.5	2.5
CaCO_3_	0.7	0.7	0.7	0.7
Alpha cellulose	3.94	11.20	3.90	2.22
Vitamins	0.75	0.75	0.75	0.75
Minerals	0.75	0.75	0.75	0.75
Y_2_O_3_	0.1	0.1	0.1	0.1
Phytase	0.05	0.05	0.05	0.05
L-carnitine	0.2	0.2	0.2	0.2
CMC	0.5	0.5	0.5	0.5
Carrageenan	0.2	0.2	0.2	0.2
Methionine	0.8	0.8	0.8	0.8
Lysine	0.21	0.21	0.21	0.21
Taurine	0.5	0.5	0.5	0.5
Total	100	100	100	100
Nutrient contents				
Protein	38.36	38.35	38.37	38.35
Lipid	14.11	14.10	14.13	14.12
Carbohydrates	27.43	27.41	27.42	27.42
P/E ratio	1.98	1.99	1.97	1.96
P available	0.78	0.77	0.79	0.77
Energy kJ g^−1^	19.35	19.36	19.34	19.36
Total phosphorus	1.38	1.39	1.37	1.38
Calcium	0.98	0.96	0.98	0.97
Ca/P	0.71	0.70	0.69	0.72
Methionine	1.39	1.37	1.38	1.37
Lysine	2.82	2.81	2.83	2.81
Arginine	2.48	2.47	2.46	2.49
Fish oil	4.55	4.54	4.56	4.54

Vitamin premix (mg kg^−1^ of diet): niacin (165); riboflavin (22); cholecalciferol (0.1); menadione (15); retinyl acetate (40); D-Ca pantothenate (102); thiamin mononitrate (45); DL-α-tocopheryl acetate (80); inositol (450); ascorbic acid (150); vitamin B12 (0.04); folic acid (10). Mineral premix (g kg^−1^): MgSO_4_·7H_2_O, 10; NaH_2_PO_4_·H_2_O, 200; CaCO_3_, 350; KH_2_PO_4_, 200; MnSO_4_·H_2_O, 2; FeSO_4_·7H_2_O, 2; Na_2_SiO_3_, 0.4; ZnSO_4_·7H_2_O, 2; CuCl_2_·2H_2_O, 1; KI, 0.1; Na_2_MoO_4_·2H_2_O, 0.5; CoCl_2_·6H_2_O, 0.1; AlCl_3_·6H_2_O, 1; NaCl, 12; and KF, 1. Triplicate analyses are used for the ultimate testing diets proximate analysis. Gross energy.

**Table 2:** Primer sequences used for real-time PCR (RT-PCR) analysis.

Target Genes	Nucleotide Sequence (5′–3′)	Product Size (bp)	Accession No: Or Publication	Amplification Efficiency
IGF-1	Forward: GTGGACGAGTGCTGCTTCCAA Reverse: GTGCCCTGCGGTACTAACCT	2299	AF030573.1	97.43
GH	Forward: GCCGTCAGCTTTCCTGATGATG Reverse: GGAGGAGACCTGCGACTACC	2469	AF502071.1	98.54
IL-1	Forward: GAATCAAGGAGGGAGACAGGA Reverse: GTAGAGGAAGACAGAGACCAA	986	JQ973887.1	98.65
TGF	Forward: TGTCTCCCCTACCCGCCGTCATC Reverse: ACCTCGCCTCCCGCTTCATCACT	3751	OQ248005.1	97.76
CCK	Forward: AGCCCAAGGCACTCTAGACA Reverse: GTTCTGGGCAGCTGTAGAGG	153	Cluster-15370.85974	97.47
CLDN1	Forward: ACTGTTAGGGTTTTTCCTGTCTC Reverse: GTGATGATGTTGTCCCCGATGTA	229	Cluster-15370.120273	98.98
Caspase-3	Forward: AGTCAGTCGAGCAGATGAAACA Reverse: GGAGAAAGCGTAGAGGAAGTC	290	Cluster-15370.106619	97.35
GPR41	Forward: CGCTGCTCGTGTTCGCTCTATG Reverse: GGTCCAGACAGGCGTTGAAGGT	398	MH381812.1	97.74
β-actin	Forward: TATCGTCATGGACTCCGGTG Reverse: TGATGTCACGCACGATTTCC	187	(Jiao et al., 2006)	98.53

Abbreviations: IGF-1, insulin-like growth factor-I; IL-1, interleukin-1; GH, growth hormone; TGF, transforming growth factor; CCK, cholecystokinin; GPR41, G protein-coupled receptor 41; CLDN-1, claudin-1; CAS-3, caspase-3.

**Table 3:** Comparison of effects of MSB, GML and TB on growth performance, feed utilization and morphometric parameters of juvenile black sea bream, *Acanthopagrus schlegelii*.

Parameters	Diets			
	Control	MSB	GML	TB
IBW	1.55 ± 0.01	1.54 ± 0.01	1.55 ± 0.01	1.54 ± 0.01
FBW	18.00 ± 0.57 ^b^	21.81 ± 1.85 ^a^	21.86 ± 1.12 ^a^	19.64 ± 1.65 ^ab^
WG (%)	1007.73 ± 35.39 ^b^	1224.12 ± 159.60 ^ab^	1324.80 ± 104.38 ^a^	1167.40 ± 107.74 ^ab^
SGR (% d^−1^)	4.24 ± 0.06 ^b^	4.52 ± 0.21 ^ab^	4.66 ± 0.11 ^a^	4.45 ± 0.15 ^ab^
MFI (g fish^−1^ day^−1^)	0.46 ± 0.07	0.48 ± 0.05	0.32 ± 0.26	0.43 ± 0.03
FCR	1.24 ± 1.21	1.23 ± 0.11	1.24 ± 0.12	1.22 ± 1.01
PER	1.85 ± 0.12 ^bc^	1.99 ± 0.12 ^ab^	2.04 ± 0.03 ^a^	1.98 ± 0.04 ^ab^
CF (g cm^−3^)	2.95 ± 0.08	2.91 ± 0.07	2.95 ± 0.05	2.95 ± 0.04
HSI (%)	1.79 ± 0.09	1.75 ± 0.05	1.83 ± 0.09	1.88 ± 0.09
IPF (%)	2.18 ± 0.22	2.06 ± 0.01	2.22 ± 0.19	2.60 ± 0.45
VSI (%)	7.38 ± 0.60	7.71 ± 0.12	7.85 ± 0.18	8.40 ± 0.75
PPV (%)	27.13 ± 1.89 ^ab^	33.92 ± 2.22 ^a^	30.34 ± 2.65 ^b^	30.01 ± 1.64 ^ab^
SR (%)	99.16 ± 1.44	99.16 ± 1.44	99.16 ± 1.44	100.00 ± 0.00

Values are presented as mean ± SD of triplicate aquaria (n = 3). Values with different superscript letters within the same row are significantly different (*p* < 0.05).

**Table 4:** Effects of different dietary levels of MSB, GML and TB on proximate composition (%) of dorsal muscle and whole body of fish on wet weight basis (n = 3).

Parameters	Diets			
	Control	MSB	GML	TB
Whole body				
Moisture	68.24 ± 0.63	68.42 ± 0.71	66.79 ± 2.79	68.39 ± 0.91
Protein	17.50 ± 0.15 ^b^	17.76 ± 0.18 ^ab^	17.85 ± 0.13 ^a^	17.67 ± 0.14 ^b^
Lipid	11.92 ± 0.12	13.39 ± 0.19	13.33 ± 0.31	12.92 ± 0.14
Ash	4.81 ± 0.31	4.74 ± 0.24	4.73 ± 0.22	4.82 ± 0.23
Dorsal muscle				
Moisture	82.68 ± 11.63	78.06 ± 7.93	82.57 ± 6.59	78.35 ± 6.57
Protein	18.52 ± 0.42	18.75 ± 0.18	18.77 ± 0.15	18.70 ± 0.04
Lipid	4.63 ± 0.16	4.66 ± 0.57	4.73 ± 0.12	4.65 ± 0.40
Ash	1.57 ± 0.13 ^a^	0.12 ± 0.06 ^b^	0.10 ± 0.02 ^b^	0.11 ± 0.14 ^b^

Values are presented as mean ± SD of triplicate aquaria (n = 3). Values with different superscript letters within the same row are significantly different (*p* < 0.05).

**Table 5:** Activities of digestive enzymes of foregut in fish that are fed with tested diets for 8 weeks (n = 3).

Parameters	Diets			
	Control	MSB	GML	TB
TP (g L^−1^)	3.99 ± 0.86	3.83 ± 0.34	4.29 ± 0.92	4.14 ± 0.70
Trypsin (U mgprot^−1^)	3032.39 ± 1374.15	2873.47 ± 295.54	3011.35 ± 613.74	2801.45 ± 474.30
Lipase (U gprot^−1^)	0.65 ± 0.14	0.93 ± 0.36	0.54 ± 0.21	0.69 ± 0.27
Amylase (U mgprot^−1^)	3.74 ± 1.48	3.15 ± 0.25	2.94 ± 1.33	3.31 ± 0.71

Values are presented as mean ± SD of triplicate aquaria (n = 3).

**Table 6:** Serum immune and antioxidant parameters of juvenile *A. schlegelii* fed experimental diets for eight weeks (n = 3).

Parameters	Diets			
	Control	MSB	GML	TB
TP (g L^−1^)	32.11 ± 4.61	36.34 ± 8.72	43.77 ± 9.83	37.67 ± 2.34
ALB (g L^−1^)	13.65 ± 3.27	11.97 ± 1.17	12.74 ± 2.15	11.46 ± 0.13
SOD (U mL^−1^)	143.34 ± 28.17	152.07 ± 38.33	173.88 ± 6.28	163.15 ± 13.47
MDA (nmol mL^−1^)	15.85 ± 2.26	14.43 ± 2.33	14.29 ± 1.80	14.81±1.54
GSH-Px (U mL^−1^)	178.24 ± 17.16 ^ab^	190.96±4.56 ^ab^	215.21±17.25 ^a^	170.38 ± 11.23 ^b^
CAT (U mL^−1^)	2.58 ± 0.26 ^b^	4.99 ± 0.70 ^a^	4.09 ± 1.34 ^a^	3.73±0.79 ^ab^
T-AOC (U mL^−1^)	1.92 ± 0.73	1.75 ± 0.16	2.05 ± 0.23	1.54 ± 0.13
LZM (U mL^−1^)	66.66 ± 17.97	71.43 ± 0.00	69.05 ± 14.86	60.71 ± 15.15
ALT (U L^−1^)	3.34 ± 0.51	2.99 ± 0.71	2.92 ± 0.92	2.89 ± 0.95
AST (U L^−1^)	10.25 ± 1.56	7.05 ± 0.97	7.52 ± 4.24	9.89 ± 1.92
T-CHO (mmol L^−1^)	10.36 ± 3.22	9.08 ± 0.63	9.62 ± 2.11	9.19 ± 0.63
IgM (mg mL^−1^)	2.04 ± 0.85	2.68 ± 0.44	1.91 ± 0.54	1.87 ± 0.78
C3 (μg mL^−1^)	276.56 ± 142.42	340.93 ± 108.76	272.96 ± 71.95	240.09 ± 38.45
C4 (μg mL^−1^)	142.63 ± 80.69	202.59 ± 61.38	127.75 ± 59.29	133.19 ± 37.71

Values are presented as mean ± SD of triplicate aquaria (n = 3). Values with different superscript letters within the same row are significantly different (*p* < 0.05).

**Table 7:** The immune and antioxidant parameters of hindgut and midgut of fish fed with the aforementioned experimental diets for a total of 8 weeks (n = 3).

Parameters	Diets			
	Control	MSB	GML	TB
Hindgut				
TP (g L^−1^)	3.74 ± 1.48	3.15 ± 0.25	2.94 ± 1.33	3.31 ± 0.71
LZM (U mL^−1^)	15.94 ± 6.13	15.95 ± 4.78	11.78 ± 5.518	15.63 ± 1.44
ALB (g L^−1^)	22.62 ± 0.14	25.31 ± 2.54	23.21 ± 0.86	22.93 ± 2.70
T-AOC (U mL^−1^)	8.21 ± 0.54 ^bc^	10.07 ± 0.45 ^a^	10.11 ± 0.47 ^a^	9.64 ± 1.04 ^b^
IgM (mg mL^−1^)	1.03 ± 0.02 ^ab^	1.45 ± 0.13 ^ab^	1.78 ± 0.03 ^a^	1.28 ± 0.04 ^ab^
Midgut				
TP (g L^−1^)	2.50 ± 0.27	2.45 ± 0.16	2.42 ± 0.04	2.47 ± 0.54
LZM (U mL^−1^)	18.62 ± 0.79 ^b^	19.29 ± 2.59 ^b^	26.03 ± 3.38 ^a^	18.79 ± 5.11 ^b^
ALB (g L^−1^)	20.32 ± 0.14	20.39 ± 0.84	20.42 ± 0.24	20.17 ± 0.64
T-AOC (U mL^−1^)	4.87 ± 1.53	5.31 ± 1.34	6.57 ± 0.43	5.00 ± 1.10
IgM (mg mL^−1^)	2.08 ± 0.06	2.30 ± 0.09	2.18 ± 0.19	2.07 ± 0.34

Values are presented as mean ± SD of triplicate aquaria (n = 3). Values with different superscript letters within the same row are significantly different (*p* < 0.05).

**Table 8:** The immune and antioxidant parameters in liver of juvenile *A. Schlegelii* fed experimental diets for eight weeks (n = 3).

Parameters	Diets			
	Control	MSB	GML	TB
TP (g L^−1^)	4.49 ± 0.71	4.46 ± 0.65	4.75 ± 0.78	4.29 ± 0.59
SOD (U mgprot^−1^)	300.06 ± 34.35	309.32 ± 43.40	315.36 ± 65.04	323.72 ± 73.45
MDA (nmol mgprot^−1^)	6.10 ± 0.27	7.33 ± 3.56	4.72 ± 0.48	5.01 ± 1.61
GSH-Px (U mgprot^−1^)	9.53 ± 1.55	8.44 ± 1.34	12.06 ± 4.61	11.79 ± 2.76
CAT (U mgprot^−1^)	22.23 ± 4.72	25.40 ± 2.79	23.54 ± 4.08	21.37 ± 1.94
T-AOC (mmol gprot^−1^)	0.15 ± 0.05	0.14 ± 0.05	0.13 ± 0.04	0.14 ± 0.04
ALT (U gprot^−1^)	14.74 ± 7.06 ^b^	20.37 ± 8.04 ^ab^	28.63 ± 5.01 ^a^	16.97 ± 2.99 ^b^
AST (U gprot^−1^)	53.17 ± 12.72	59.08 ± 0.51	65.99 ± 7.02	66.26 ± 23.45

Values are presented as mean ± SD of triplicate aquaria (n = 3). Values with different superscript letters within the same row are significantly different (*p* < 0.05).

**Table 9:** Effect of different dietary levels of MSB, GML and TB on structure of fore intestinal mucosa in juvenile black sea bream, *A. schlegelii* (n = 3).

Parameters	Diets			
	Control	MSB	GML	TB
VH (μm)	432.82 ± 3.54 ^c^	502.49 ± 4.48 ^a^	504.83 ± 10.49 ^a^	483.04 ± 17.29 ^b^
CD (μm)	84.09 ± 16.31 ^b^	97.43 ± 2.32 ^ab^	98.36 ± 2.51 ^a^	95.59 ± 2.15 ^ab^
VH/CD (μm)	7.08 ± 0.25 ^b^	7.98 ± 0.88 ^ab^	8.73 ± 0.04 ^a^	7.87±0.34 ^ab^
GC/VH	15.18 ± 1.15 ^b^	24.23 ± 2.10 ^a^	26.35 ± 2.25 ^a^	23.70 ± 2.10 ^a^

Values are presented as mean ± SD of triplicate aquaria (n = 3). Values with different superscript letters within the same row are significantly different (*p* < 0.05). Note: VH, villus height; CD, crypt density; VH/CD, villus height/crypt density; Number of goblet cells/villus height GC/VH.

**Table 10:** Overview of sequencing data on the intestinal microbiota of black sea bream (*Acanthopagrus schlegelii*) fed experimental diets.

Parameters	Diets			
	Control	MSB	GML	TB
Raw tags	79,583	83,191	85,738	81,348
Valid tags	70,274	78,153	79,137	72,089
Coverage	1.00	1.00	1.00	1.00

**Table 11:** Analysis of alpha diversity indices and coverage estimates of the intestinal microbiota in black sea bream (*Acanthopagrus schlegelii*) fed experimental diets.

Index	Diets			
	Control	MSB	GML	TB
Observed species	191.58 ± 2.26 ^d^	232.31 ± 3.40 ^b^	285.310 ± 1.67 ^a^	214.69 ± 3.90 ^c^
Shannon	3.51 ± 0.01 ^c^	4.23 ± 0.12 ^b^	4.64 ± 0.12 ^a^	4.18 ± 0.03 ^b^
Simpson	0.93 ± 0.02 ^b^	1.00 ± 0.03 ^a^	1.03 ± 0.01 ^a^	0.98 ± 0.02 ^ab^
Chao1	230.67 ± 2.76 ^b^	258.16 ± 9.17 ^a^	265.81 ± 8.91 ^a^	251.72 ± 2.07 ^a^

Different letters on bars show significant difference (*p* < 0.05). a, ab, b letters indicate statistical significance between the diet groups. a and ab are not significantly different, while a vs. b show a significant difference.

**Table 12:** The composition of microbiota at the phylum level in the intestine of *Acanthopagrus schlegelii* (%).

Phylum	Diets			
	Control	MSB	GML	TB
P__Proteobacteria	80.15 ± 3.56 ^c^	87.92 ± 2.94 ^ab^	92.095 ± 4.81 ^a^	83.28 ± 1.29 ^bc^
p__Cyanobacteria	17.07 ± 0.31 ^a^	16.05 ± 1.57 ^ab^	14.49 ± 0.49 ^b^	15.09 ± 0.50 ^b^
p__Actinobacteria	7.05 ± 0.69	6.01 ± 2.73	7.15 ±1.79	5.63 ± 0.28
p__Firmicutes	5.47 ± 0.49 ^b^	5.90 ± 0.11 ^ab^	7.39 ± 0.98 ^a^	6.74 ± 0.96 ^ab^
p__Acidobacteria	2.24 ± 0.95	2.57 ± 0.96	2.90 ± 0.31	2.85 ± 0.20
p__Bacteroidetes	1.11 ± 0.54	1.19 ± 1.00	1.39 ± 1.08	1.22 ± 1.06

Values are presented as mean ± SD of triplicate aquaria (n = 3). Values with different superscript letters within the same row are significantly different (*p* < 0.05).

**Table 13:** The intestinal microbiota composition of *Acanthopagrus schlegelii* was analyzed at the class level (%).

Class	Diets			
	Control	MSB	GML	TB
c__Alphaproteobacteria	34.82 ± 2.68 ^b^	36.19 ± 3.26 ^ab^	41.10 ± 1.39 ^a^	39.85 ± 1.96 ^ab^
c__Betaproteobacteria	23.82 ± 0.40 ^c^	24.39 ± 0.97 ^bc^	27.53 ± 0.94 ^a^	25.47 ± 0.63 ^b^
c__Gammaproteobacteria	13.89 ± 0.60 ^b^	14.38 ± 0.46 ^ab^	15.14 ± 0.76 ^a^	14.61 ± 0.11 ^ab^
c__Chloroplast	3.84 ± 0.44	3.56 ± 0.79	6.26 ± 2.36	4.16 ± 0.97
c__Actinobacteria	3.69 ± 0.85	4.41 ± 1.33	4.64 ± 0.31	3.77 ± 0.85
c__Clostridia	1.48 ± 0.16 ^b^	1.56 ± 0.04 ^b^	1.96 ± 0.04 ^a^	1.67 ± 0.24 ^ab^
c__Acidobacteria_Gp6	1.02 ± 0.46	0.80 ± 0.22	1.06 ± 0.41	0.79 ± 0.22
c__Sphingobacteriia	0.55 ± 0.37	0.49± 0.28	0.76 ± 0.23	0.68 ± 0.48
c__Bacilli	0.16 ± 0.05 ^b^	0.78 ± 0.44 ^ab^	1.21 ± 0.42 ^a^	0.26 ± 0.16 ^b^

Values are presented as mean ± SD of triplicate aquaria (n = 3). Values with different superscript letters within the same row are significantly different (*p* < 0.05).

**Table 14:** The composition of microbiota at the family level in the intestine of *Acanthopagrus schlegelii* (%).

Family	Diets			
	Control	MSB	GML	TB
f__Caulobacteraceae	12.99 ± 2.36	17.69 ± 4.67	17.91 ± 3.77	15.26 ± 3.28
f__Xanthomonadaceae	1.34 ± 0.30	1.42 ± 0.39	1.88 ± 0.84	1.39 ± 0.22
f__Sphingomonadaceae	11.66 ± 0.30 ^b^	13.71 ± 1.05 ^ab^	15.02 ± 1.76 ^b^	12.85 ± 0.53 ^ab^
f__Burkholderiaceae	8.03 ± 3.00	11.16 ± 2.69	11.47 ± 2.36	11.05 ± 1.59
f__Oxalobacteraceae	12.79 ± 1.73	14.66 ± 2.92	17.42 ± 4.52	13.24 ± 3.33
f__Chloroplast	7.29 ± 0.48 ^c^	9.61 ± 0.33 ^ab^	10.44 ± 0.54 ^a^	9.41 ± 0.47 ^b^
f__Rhodocyclaceae	6.84 ± 0.44	8.29 ± 1.89	10.54 ± 2.11	8.28 ± 2.45
f__Comamonadaceae	6.39 ± 1.37	5.67 ± 1.68	7.93 ± 1.43	5.18 ± 1.58
f__Moraxellaceae	2.04 ± 0.69	2.95 ± 0.64	4.41 ± 2.47	2.29 ± 0.99
f__Pseudomonadaceae	4.10 ± 0.53	4.60 ± 0.17	5.27 ± 1.32	5.08 ± 0.91
f__Hyphomicrobiaceae	1.93 ± 0.07 ^c^	2.12 ± 0.04 ^b^	2.22 ± 0.04 ^a^	2.09 ± 0.01 ^b^
f__Nocardiaceae	0.50 ± 0.09 ^b^	0.67 ± 0.32 ^b^	1.77 ± 0.72 ^a^	0.62 ± 0.26 ^b^
f__Bradyrhizobiaceae	0.50 ± 0.09 ^b^	0.67 ± 0.32 ^ab^	1.07 ± 0.09 ^a^	0.740 ± 0.18 ^ab^
f__Iamiaceae	0.89 ± 0.07 ^ab^	0.94 ± 0.34 ^ab^	1.11 ± 0.21 ^a^	0.68 ± 0.05 ^b^
f__Clostridiaceae_1	0.54 ± 0.13^c^	0.93 ± 0.09 ^ab^	0.97 ± 0.02 ^a^	0.80 ± 0.02 ^b^
f__Gp6	0.60 ± 0.31	0.65 ± 0.46	0.78 ± 0.13	0.52 ± 0.03

Values are presented as mean ± SD of triplicate aquaria (n = 3). Values with different superscript letters within the same row are significantly different (*p* < 0.05).

## Data Availability

The original contributions made in this study are contained within the article; any additional inquiries can be directed to the corresponding authors.
